# Parallel and non-parallel morphological divergence among foraging specialists in European whitefish (*Coregonus lavaretus*)

**DOI:** 10.1002/ece3.562

**Published:** 2013-04-25

**Authors:** Anna Siwertsson, Rune Knudsen, Colin E Adams, Kim Præbel, Per-Arne Amundsen

**Affiliations:** 1 Department of Arctic and Marine Biology, University of TromsøN-9037 Tromsø, Norway; 2 Scottish Centre for Ecology and the Natural Environment, University of GlasgowRowardennan, Glasgow G63 0AW, Scotland

**Keywords:** Adaptive radiation, ecological speciation, geometric morphometrics, natural selection, polymorphism, profundal specialization

## Abstract

Parallel phenotypic evolution occurs when independent populations evolve similar traits in response to similar selective regimes. However, populations inhabiting similar environments also frequently show some phenotypic differences that result from non-parallel evolution. In this study, we quantified the relative importance of parallel evolution to similar foraging regimes and non-parallel lake-specific effects on morphological variation in European whitefish (*Coregonus lavaretus*). We found evidence for both lake-specific morphological characteristics and parallel morphological divergence between whitefish specializing in feeding on profundal and littoral resources in three separate lakes. Foraging specialists expressed similar phenotypes in different lakes in both overall body shape and selected measured morphological traits. The morphology of the two whitefish specialists resembled that predicted from other fish species, supporting the conclusion of an adaptive significance of the observed morphological characteristics. Our results indicate that divergent natural selection resulting from foraging specialization is driving and/or maintaining the observed parallel morphological divergence. Whitefish in this study may represent an early stage of divergence towards the evolution of specialized morphs.

## Introduction

Populations that experience different selective environments often diverge in morphological, physiological, behavioral, and life-history traits (Skúlason and Smith 1995; Schluter 2000; Bernatchez 2004). This adaptive population divergence often produces parallel patterns of divergence in independent lineages (e.g., species) inhabiting similar environments. Although parallel evolution has been reported for a wide range of taxa (e.g., Jones et al. 1992; Eroukhmanoff et al. 2009; Losos 2009; Langerhans 2010), these independent populations inhabiting similar environments also frequently show some phenotypic differences, resulting from non-parallel evolution (e.g., Berner et al. 2010; Rosenblum and Harmon 2011; Kaeuffer et al. 2012). One form of population divergence that often shows strong evidence of parallel phenotypic divergence is resource polymorphisms.

Resource polymorphisms, that is when multiple discrete phenotypes within a population utilize different resources, have been described from several vertebrate taxa, including fish, birds, amphibians, and mammals (Wimberger 1994; Skúlason and Smith 1995; Smith and Skúlason 1996). The occurrence of discrete morphological variation with differential resource use implies a general close association between ecological and morphological traits. Expressed morphology is known to directly affect resource use performance (e.g., Arnold 1983; Wainwright 1994, 1996) and thus ultimately fitness. Thus, morphology associated with resource use is likely to be under strong natural selection (e.g., Wainwright 1994). In this study we quantified the relative importance of parallel and non-parallel morphological divergence in populations where we previously have identified an incipient polymorphism based on resource use (Siwertsson et al. 2013).

Whitefish (*Coregonus* sp.) has a circumpolar distribution in lakes in the northern hemisphere, and is known to express resource polymorphism, especially along the pelagic (open water) and benthic axis (Skúlason and Smith 1995; Smith and Skúlason 1996). In northern Fennoscandia the European whitefish (*Coregonus lavaretus*) is a highly polymorphic fish species with up to five sympatric morphs (Svärdson 1979; Bergstrand 1982; Østbye et al. 2005b). The most commonly occurring morph pair in northern Fennoscandia shows a close association between resource use and morphological traits (Kahilainen and Østbye 2006; Harrod et al. 2010; Kahilainen et al. 2011). Morphs from a typical pair comprise a specialist zooplanktivore, which forages in the pelagic (open water) zone and is typified by many and densely packed gill rakers (called the densely rakered morph), and a morph displaying shorter and fewer gill rakers and larger body size (the large sparsely rakered (LSR) morph), specializing on benthic living macro-invertebrates (Amundsen 1988; Amundsen et al. 2004a,b; Kahilainen et al. 2004; Siwertsson et al. 2010).

This study is based on the recently recorded more subtle differentiation in foraging specialization within the whitefish exhibiting large body size and sparse gill rakers (LSR morph) in three different lakes in northern Fennoscandia (Siwertsson et al. 2013). Individuals specializing either on profundal (deep water benthic) or littoral (shallow water benthic) habitat and prey resources were detected from clear differences both in recent (stomach contents) and long-term resource use (based on stable isotope ratios of carbon and nitrogen). Clear ecological behavioral divergence was accompanied by small but significant differences in gill raker number, an adaptive morphological trait with foraging efficiency consequences (Sanderson et al. 2001; Kahilainen et al. 2011). Genetic analysis also showed that the two foraging specialists comprised two partially separate gene pools within each lake (F_ST_: 0.014–0.024) (Siwertsson et al. 2013). There is evidence that the profundal specialization in whitefish has arisen independently in different lakes within a nearby watercourse (Præbel et al. in review).

The deep profundal habitat in lakes is a very different environment compared to the shallow littoral areas. The littoral offers a complex physical environment, with a range of different substratum types and submerged vegetation. Temperature and light conditions vary daily and seasonally, and both food resources and predators are typically diverse. In contrast, the profundal habitat is considerably more uniform, consisting of fine sediments with no vegetation, consistently low light conditions, and minimal variation in temperature. Food resources are scarce and typically consist of small invertebrates partly buried in soft sediments. Fish species specializing in profundal resources often exhibit small body size with deep body form, large head compared to body size, long snout, ventrally positioned large mouth, dorsally positioned large eyes, large pectoral and dorsal fins and sometimes a reduced or malfunctioning swimbladder (Turgeon et al. 1999; Klemetsen et al. 2002; Kahilainen and Østbye 2006; Zimmerman et al. 2006; Harrod et al. 2010; Genner and Turner 2012; Gowell et al. 2012).

The general objective of this study was to test if the presumed common selection pressure imposed by the physical environment and resource use, operating on these previously identified profundal and littoral resource specialists, has resulted in parallel morphological evolution. Specifically, we predicted that the morphology of foraging specialists would be similar in each of the three lakes, indicating that similar selection pressures are having a similar effect on morphological traits.

## Materials and Methods

### Study area and sampling

Large sparsely rakered whitefish were sampled from three lakes situated in the Alta-Kautokeino watercourse in the sub-arctic region of northern Norway. The lakes are oligotrophic with some humic impact from the surrounding tundra (Table 1). They are of varying size, but all have well-developed littoral (with >1% of surface light levels) and profundal (<1% of surface light levels) zones (Table 1). The lakes are in vicinity of each other and it is highly likely that they were all subject to the same postglacial and colonization processes (Østbye et al. 2005a). Lake Lahpojavri (LP) (69.25°N, 23.78°E) is situated in a different tributary isolated from the other two by waterfalls. Migration from Lake Suopatjavri (SU) (68.93°N, 23.09°E) to the downstream Lake Vuolgamasjavri (VG) (69.14°N, 23.36°E) is probable, while upstream migration is theoretically possible but less likely due to the presence of rapids. Fish were sampled during late August – early September in 2007 or 2008 from littoral (1–8 m) and profundal (18–35 m) habitats using multi-mesh survey gillnets (length 40 m, height 1.5 m) with mesh sizes of 10, 12.5, 15, 18.5, 22, 26, 35, and 45 mm (5 m of each) set overnight. The 265 fish caught in littoral and profundal habitats were measured (fork length) to the nearest millimeter. More details about the sampling procedures can be found in Siwertsson et al. (2013).

**Table 1:** Characteristics of the three study lakes

	Lahpojavri	Suopatjavri	Vuolgamasjavri
Surface area (km^2^)	8.1	2	1.2
Perimeter (km)	46.3	10.5	19.7
Maximum depth (m)	36	25	30
Mean depth (m)	8.7	8.2	14.9
Littoral^1^ (%)	58	61	27
Profundal^1^ (%)	42	39	73
Total phosphorus (mg l^−1^)	5	9	–2
Total nitrogen (mg l^−1^)	202	243	–2
Secchi depth (m)	4	4	4.5

1 Availability of littoral and profundal habitats are measured in percent of lake surface area.

2 Measures of total phosphorus and total nitrogen are not available for Vuolgamasjavri.

Following Siwertsson et al. (2013), we used the capture habitat where the individual was caught as a conservative proxy for resource specialization. For these lakes, habitat was shown to be a good indicator of long-term resource use (measured by analyses of stable isotope ratios of carbon and nitrogen), and 79–100% of the individuals were correctly classified to a diet specialist group based on habitat alone. Differences between fish from littoral and profundal habitats in resource utilization, gill raker number, and neutral genetic markers based on data from Siwertsson et al. (2013) are summarized in Table 2.

**Table 2:** Differences between LSR whitefish caught in littoral (Lit) and profundal (Prof) habitats based on Siwertsson et al. (2013), and sample size (*N*) for the morphometric analyses in this study

						Stomach contents			
							
Lake	Habitat	*N*	*F*_ST_^1^	SI^2^	Diet^3^	Prof	Lit	Pel	Gill rakers^4^
Lahpojavri			0.024	5.2	0.13				
	Lit	44				12	**84**	4	26.7
	Prof	15				**99**	1	0	24.9
Suopatjavri			0.019	4.6	0.29				
	Lit	40				6	39	**55**	27.8
	Prof	15				**72**	5	23	25.3
Vuolgamasj			0.014	4.7	0.26				
	Lit	43				9	**47**	44	25.3
	Prof	36				**73**	13	14	23.4

Genetic differentiation (*F*_ST_), difference in centroid location of stable isotope ratios of carbon and nitrogen (SI), and diet similarity (Diet) between fish from the two habitats. Stomach contents (%) were divided into profundal (Prof), littoral (Lit), and pelagic (Pel) prey items, and the most important prey group is in boldface.

1 Based on 16 neutral microsatellite loci. All comparisons were statistically significant (*P* < 0.05).

2 All comparisons were statistically significant (*P* ≤ 0.001).

3 Schoeners index based on stomach contents. Values >0.6 are generally interpreted as biologically significant similarities.

4 Mean number of gill rakers. All comparisons were statistically significant (P ≤ 0.01).

### Morphological analyses

**Figure 1:**
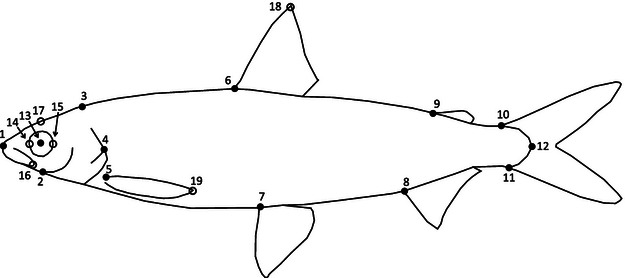
Illustration of landmark positions used in geometric morphometrics and measurements of morphological traits. The interlandmark distance between 1 and 12 was used as a measure of standard length for the size correction of trait measurements. Only landmarks 1 – 13 (filled symbols) were included in the geometric morphometric analyses of body shape.

The left side of the fish was photographed with a digital camera (Nikon Coolpix 5400), and 19 landmarks were digitized on 193 good quality pictures of fish using TPSDig2 v2.16 (Fig. 1) (Rohlf 2010). Ten morphological traits of possible functional importance were measured as the distance between specific landmark pairs (Table 3). These traits were selected based on significance of differences between littoral and profundal morphs of Arctic charr (*Salvelinus alpinus*), whitefish, and lake trout (*Salvelinus namaycush*) (Klemetsen et al. 2002; Kahilainen and Østbye 2006; Zimmerman et al. 2006). The predicted direction of the divergence for each trait based on previous studies of littoral and profundal foraging specialists in fish is shown in Table 3. Four of these traits (eye diameter, snout length, maxilla length, and dorsal fin length) have been shown to have a genetic basis in Arctic charr morphs (Klemetsen et al. 2002).

**Table 3:** Ten morphological traits of possible adaptive value, measured as the distance between specific landmark pairs (Fig. 2)

Morphological trait	Landmarks	Expected direction	Observed direction		
Eye diameter	14–15	P > L^1^^,^^2^	P > L	*P* < 0.001	***
Snout length	1–14	P > L^1^^,^^2^	P = L	*P* = 0.06	NS
Maxilla length	1–16	P > L^1^^,^^2^	P > L	*P* < 0.001	**
Head length	1–4	P > L^1^^,^^2^	P > L	*P* < 0.001	***
Head depth	2–17	P > L^2^^,^^3^	P > L	*P* < 0.001	***
Body depth anterior	6–7	P > L^1^^,^^2^	P > L	*P* < 0.001	**
Body depth posterior	8–9	L > P^1^	P = L	*P* = 0.59	NS
Caudal peduncle depth	10–11	L > P^1^, P = L^2^, P > L^3^	P ≥ L	*P* < 0.05	NS
Dorsal fin length	6–18	P > L^2^, L > P^3^	P > L	*P* < 0.001	***
Pectoral fin length	5–19	P > L^1^^,^^2^^,^^3^	P > L	*P* < 0.001	***

These traits were selected based on significance of differences between littoral and profundal morphs of other salmonid fish species. The expected and observed directions of differences are indicated for each trait (P: profundal, L: littoral). *P*-values for the observed differences between littoral and profundal specialists are based on t-tests of each size-corrected trait and stars indicate significance levels after Bonferroni correction (* *P* < 0.05, ** *P* < 0.01, *** *P* < 0.001, NS: *P* > 0.05).

1 Klemetsen et al. (2002) Evidence for genetic differences in the offspring of two sympatric morphs of Arctic charr. J Fish Biol 60:933-950.

2 Kahilainen and Østbye (2006) Morphological differentiation and resource polymorphism in three sympatric whitefish *Coregonus lavaretus* (L.) forms in a subarctic lake. J Fish Biol 68:63–79.

3 Zimmerman et al. (2006) Phenotypic diversity of lake trout in Great Slave Lake: differences in morphology, buoyancy, and habitat depth. Trans Am Fish Soc 135:1056–1067.

As the morphological measures were correlated to individual body length, all traits were allometrically scaled to the average standard length (Fig. 1) of all whitefish (21.34 cm) (Senar et al. 1994). First, all traits were log_10_-transformed to reduce heterogeneity of variances. Then, we calculated the common slope (*b*) for each trait (log_10_-transformed) using an ANCOVA model with all combinations of lakes and foraging specialists (six groups) and standard length as explaining variables. The slope was used to standardize morphological trait measurements to the mean size using the allometric growth formula:


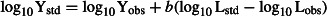
(1)

where Y_std_ is the size standardized trait value, Y_obs_ is the measured trait value, L_std_ is the mean body length of whitefish, and L_obs_ is the measured body length. The performance of the size-correction method was checked by linear regressions of each trait and body length, which were all non-significant. For multivariate analyses of the combination of all linear trait measurements, we used the log_10_-transformed size standardized trait values (log_10_Y_std_) throughout.

Body shape was quantified using landmark based geometric morphometrics, based on 13 landmarks (Fig. 1) (Adams et al. 2004; Zelditch et al. 2004). To compare shape differences only, effects of size, position, and orientation were removed from landmark configurations by Procrustes superimposition using MorphoJ v.1.03d (Klingenberg 2011). The standardized landmark coordinates, Procrustes coordinates, were used as shape variables in analyses of body shape.

### Statistical analyses

General differences in each of the ten measured linear traits between littoral and profundal foraging specialists were statistically tested using t-tests with Bonferroni corrections to adjust significance levels.

Two multivariate methods were used to identify patterns of morphological variation between the predefined foraging specialists. A Principal Component Analysis (PCA) was performed on the shape variables and on the linear measurements to explore the major axes of morphological variation among individuals. Two-way analysis of variance (ANOVA) was used to test for differences between lakes, foraging specialist groups and their interaction in the most important first three Principal Components. Canonical Variate Analyses (CVA) complemented the PCAs in using the predefined group information to maximize between-group variation relative to within-group variation. The CVAs were performed on the shape variables and the linear traits separately, and were used to quantify and visualize differences in morphology between the two foraging specialist groups and between lakes. The accuracy of the discrimination functions was assessed by leave-one-out cross-validation. Multivariate morphological differences (for shape variables and linear traits separately) were statistically examined using Hotelling's *T*^2^ tests between foraging groups, and one-way multivariate analysis of variance (MANOVA) between lakes. To illustrate body shape features associated with the different PC and CV axes, in the analyses using shape variables, we used thin-plate splines (TPS) to produce transformation grids representing positive and negative deviations from the mean shape. Transformation grids were generated using MorphoJ v.1.03d (Klingenberg 2011). Sexual dimorphism was not observed in either body shape (Hotelling's *T*^2^_22,170_ = 14.73, *P* = 0.92) or the measured morphological traits (Hotelling's *T*^2^_10,182_ = 1.23, *P* = 0.27), and analyses were performed on both sexes combined.

To evaluate the relative importance of parallel and non-parallel (divergent) evolutionary effects on morphology, we followed the logical framework of Langerhans and DeWitt (2004). A parallel morphological response was deemed to occur where there was a similar morphological divergence across foraging specialists between different lakes. A non-parallel response was defined as when there were lake-specific differences (or lake-foraging specialization interactions). To test this we performed a two-way MANOVA, separate for body shape and linear trait measurements, with lake, foraging specialization, and their interaction as factors predicting morphology. To evaluate the relative importance of the three factors we estimated effect sizes using Wilk's partial η^2^ (partial variance; multivariate approximation of SS_effect_/(SS_effect_ + SS_error_), see Appendix of Langerhans and DeWitt 2004). As Procrustes coordinates are not free from allometric effects (Klingenberg 1996), a multivariate analysis of covariance (MANCOVA) was used for the analyses of body shape. The response variables were all the Principal Components (PCs; from the PCA using shape variables), and centroid size served as the covariate controlling for multivariate allometry. Canonical variate scores from these two-way MAN(C)OVAs were extracted using the candisc package v.05-21 by M. Friendly and J. Fox in R.

The CVA describing body shape differences between the three lakes was performed in PAST v.2.15 (Hammer et al. 2001) using all the PCs with non-zero eigenvalues to ensure that the degrees of freedom were correctly computed from the Procrustes coordinates. Other analyses using shape variables were performed in MorphoJ v.1.03d (Klingenberg 2011). All other statistical analyses were performed in the R statistical environment (R Development Core Team 2011).

## Results

### Overall body size and individual trait measurements

Whitefish body size (fork length) was similar in littoral and profundal habitats (two-way ANOVA: F_1,259_ = 0.460, *P* = 0.50), but differed between lakes (F_2,259_ = 7.485, *P* < 0.001). Whitefish from SU were larger (mean ± SD: 25.4 ± 6.7 cm) than in LP (22.2 ± 4.2 cm) and VG (22.7 ± 5.7 cm) (Tukey's pairwise HSD tests: SU-LP and SU-VG *P* < 0.01, LP-VG *P* = 0.77).

Significant differences between littoral and profundal foraging specialists were found in seven of the ten selected linear traits (Table 3). In all these traits, profundal specialists expressed larger values compared with littoral specialists, which was also expected based on previous studies (Table 3).

### Major axes of morphological variation (PCAs)

**Figure A1:**
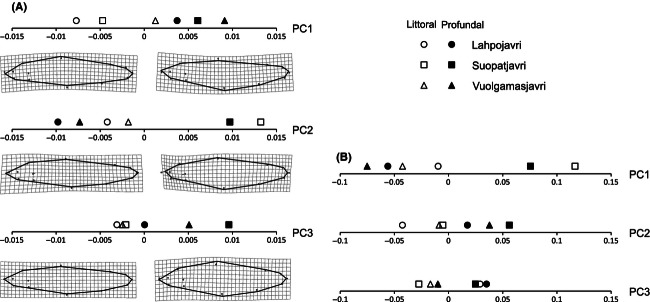
Mean values for littoral and profundal resource specialists in each of the three lakes along the first three PC-axes from PCA of a) shape variables and b) ten linear morphological traits. Shape changes along the PC-axes are illustrated (a) using thin-plate spline transformations of the mean shape in the most extreme populations relative to the overall mean shape. Shape changes are magnified 3 times for easier interpretation. The landmark vectors show the relative magnitude of change in the location of each landmark, with the points representing the overall mean shape, and lines pointing in the direction of the lake-specific morphology. Solid lines between ends of vectors are drawn to aid interpretation. The importance of individual linear traits on each PC-axes in b) is presented in Appendix Table A2.

In the PCA of body shape, the first PC (28.5% of total variation) was mainly associated with bending of the fish body, which is an unwanted effect occurring during the photographing (Fig. A1). The second and third PCs described body shape variation independent of bending. High values of the second PC (17.0%) were associated with shorter head and caudal region, and deeper body form (Fig. A1). A two-way ANOVA revealed significant differences between both lakes and foraging specialists on this PC axis (Table A1). Profundal specialists had significantly higher values on the third PC (14.7%), which was associated with more robust body and head, and down-facing tip of the snout (Fig. A1 and Table A1).

Using size-corrected linear measurements, the first three PCs explained 82% of the total morphological variation. The first PC (59.2%) was affected by smaller head characteristics (smaller eye, shorter snout, maxilla, and head) (Table A2). The second PC (14.7%) was mainly affected by longer fins (dorsal and pectoral), and the third PC (8.2%) by larger eye and shorter maxilla length (Table A2). There were significant differences both between lakes and foraging specialists in all three PC axes (Table A1, Fig. A1). However, based on the F-values differences between littoral and profundal foraging specialists were best described by the second PC, while between lake differences were described by the first and second PC (Table A1, Fig. A1).

### Morphological differences between littoral and profundal specialists

**Figure 2:**
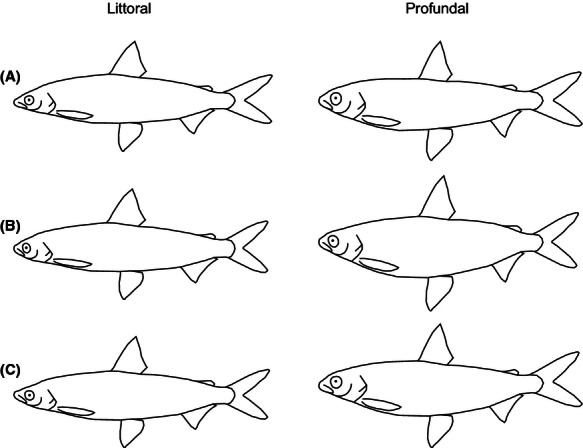
Illustration of body shape differences between whitefish specializing on littoral and profundal resources in (A) LP, (B) SU, and (C) VG. Figures represent thin-plate spine transformations of the mean shape of each foraging specialist group from the consensus shape in each lake, and are magnified 3 times for easier interpretation. Note that the size of fins and eye was not included in the analyses of body shape and should not be interpreted from the illustrations.

**Figure 3:**
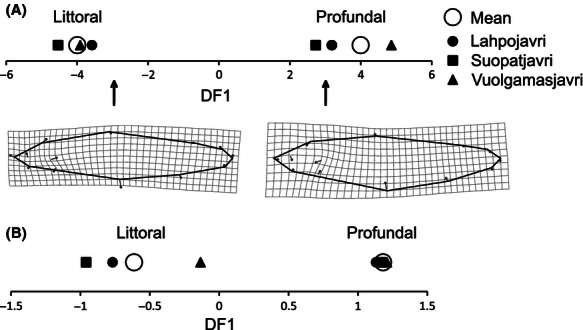
Morphological differentiation between whitefish specializing on littoral and profundal resources. (A) Body shape variation is described by the discriminant function (DF1) from the discrimination analysis of shape *versus* foraging specialization, and the shapes at the position of the arrows are illustrated using thin-plate spline transformations relative to overall mean shape. The landmark vectors show the relative magnitude of change in the location of each landmark, with the points representing the overall mean shape, and lines pointing in the direction of littoral and profundal morphology, respectively. Solid lines between ends of vectors are drawn to aid interpretation. (B) Variation in linear morphological traits is described by the discriminant function (DF1) from the discrimination analysis of all ten linear traits *versus* foraging specialization. Importance of individual traits on DF1 is presented in Table 4.

The discrimination analyses revealed that across all lakes 90.7% of all fish were correctly assigned to their foraging specialization based on body shape, and 83.4% based on linear trait measurements (cross-validated values). This indicates that fish specializing on similar resources had similar morphology, irrespective of the lake of origin. The body shape of littoral and profundal specializing fish differed significantly (Hotelling's *T*^2^ = 346.98, *P* < 0.001), with a difference measured as Procrustes distance of 0.015. Littoral specialists had a more slender body shape and smaller head compared with profundal specialists, which were characterized by a deeper body and head, with the eye more dorsally positioned (Figs. 2, 3).

The linear trait measurements were also significantly different between the foraging specialist groups (Hotelling's *T*^2^ = 13.33, *P* < 0.001). Based on the loadings on the discriminant axis (Table 4) and *t*-tests of individual size-corrected trait measurements (Table 3) profundal specialists had longer heads, larger eyes, and longer pectoral fins compared with littoral specialists (Fig. 3). Profundal specializing fish also had larger anterior compared to posterior body depth, that is, had an enlarged anterior part of the body. Altogether seven of the ten measured traits were significantly different between littoral and profundal specialists in the expected direction based on the literature (Table 3).

**Table 4:** Loadings (importance) of the different morphological traits on the discriminant function axes for morphological differences between whitefish specializing on littoral and profundal resources, and from different lakes. The three most important traits on each axis are indicated by boldface

Morphological trait	Foraging specialization	Lake (CV1)	Lake (CV2)
Eye diameter	6.60	−13.32	−9.77
Snout length	−10.01	−15.37	−1.43
Maxilla length	1.71	8.96	**22.09**
Head length	**26.02**	**15.91**	**15.87**
Head depth	3.82	−0.42	−**14.31**
Body depth anterior	**15.00**	**16.31**	8.35
Body depth posterior	−12.20	**17.81**	2.11
Caudal peduncle depth	2.45	−6.77	−8.79
Dorsal fin length	2.75	8.83	10.51
Pectoral fin length	**14.07**	−8.20	4.42

### Morphological differences between lakes

**Figure 4:**
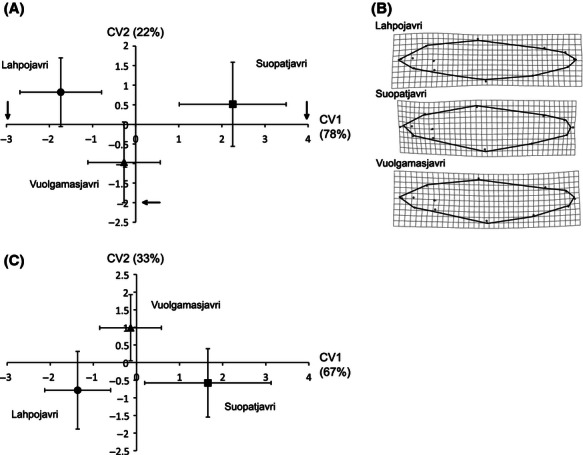
Differences in morphology between whitefish from the three different lakes based on Canonical Variate Analyses (CVA) of (A) body shape *versus* lake, and (C) ten linear traits *versus* lake (mean ± SD for each lake). Arrows in (A) indicate the positions of shapes in (B). Body shape variation (B) is described by the discriminant functions from the CVA, illustrated using thin-plate spline transformations relative to overall mean shape. The landmark vectors show the relative magnitude of change in the location of each landmark, with the points representing the overall mean shape, and lines pointing in the direction of the lake-specific morphology. Solid lines between ends of vectors are drawn to aid interpretation. The importance of individual linear traits on CV1 and CV2 in (C) is presented in Table 4.

Our classification of all fish to lake origin correctly assigned 82.9% based on body shape, and 76.2% based on measured morphological traits. Fish body shape was significantly different between all three lakes (MANCOVA (covariate: centroid size): Wilk's lambda = 0.177, df = 44, 336, *P* < 0.001). The magnitude of shape differences between lakes, as measured by Procrustes distances, was larger between SU and the other two lakes (LP: 0.020, VG: 0.018) than between LP and VG (0.012). Fish from SU generally had a deeper body and markedly shorter heads and caudal peduncles than in the other two lakes (Figs. 2, 4). Fish from VG had the most robust overall shape, with deep body forms and large heads.

Whitefish from the three lakes were also different in the measured morphological traits (Fig. 4, MANOVA: Wilk's lambda = 0.248, df = 20, 362, *P* < 0.001). High values on the most important first discriminant axis (CV1) were associated with smaller eye, shorter snout, longer head, and deeper body form (Table 4). Fish from SU had high values of CV1 (mean ± SD: 1.66 ± 1.47), VG intermediate (−0.14 ± 0.72), and LP had low values (−1.36 ± 0.76) (Fig. 4).

### Parallel and non-parallel morphological divergence

**Figure 5:**
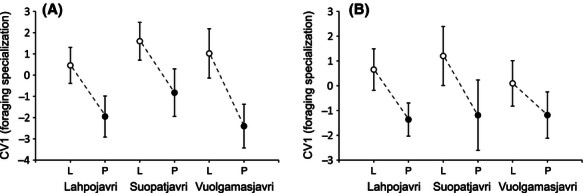
Canonical scores discriminating between littoral (L) and profundal (P) specialist groups, extracted from MAN(C)OVAs including the effects of foraging specialization, lake and their interaction on (A) body shape and (B) linear trait measurements.

The two-way MANCOVA used to estimate the relative importance of foraging specialization, lake, and their interaction on body shape variation revealed significant effect of centroid size (*F* = 14.63, df = 22, 165, *P* < 0.001), indicating multivariate allometry. We found significant effects for all factors of primary interest on both body shape and linear trait measurements, indicating both parallel and non-parallel morphological evolution (Table 5). Lake was always the most important term with partial variances of 52% for body shape and 98% for linear trait measurements. The partial variance explained by parallel evolution to similar foraging specializations was 38% using body shape and 63% using linear trait measurements (Table 5). However, the morphological divergence between littoral and profundal specialists was not of the same magnitude in all lakes, which resulted in significant interaction terms (Fig. 5, Table 5).

**Table 5:** Results from two-way MANCOVA of body shape and two-way MANOVA of linear trait measurements comparing the relative importance of parallel (foraging specialization; Spec.) and non-parallel (Lake, and Spec. × Lake interaction) effects on morphology

	Effect	Wilk's Lambda	df	*P*-value	Partial variance^1^ [%]	Relative variance^2^ [%]
Body shape	Spec.	0.624	22, 165	<0.001	37.6	72.7
	Lake	0.233	44, 330	<0.001	51.7	100
	Spec. × Lake	0.422	44, 330	<0.001	35.0	67.7
Trait values	Spec.	0.567	10, 178	<0.001	63.2	64.4
	Lake	0.299	20, 356	<0.001	98.0	100
	Spec. × Lake	0.630	20, 356	<0.001	51.9	52.9

1 Partial variance explained was estimated using Wilk's partial η^2^.

2 Relative variance represents partial variance for a given factor divided by the maximum partial variance in the model (i.e., for lake).

## Discussion

Parallel phenotypic evolution occurs when independent evolutionary lineages evolve similar traits in response to similar selective regimes, and has been documented for a wide range of animals (e.g., Jones et al. 1992; Eroukhmanoff et al. 2009; Losos 2009; Langerhans 2010). In this study, we found evidence for parallel morphological divergence between profundal and littoral resource specialists in three populations of European whitefish. This parallelism was found in both overall body shape and selected morphological traits. Evidence suggests that specialization on littoral and profundal resources developed in sympatry within these lakes. First, migration between lakes is likely to be low or absent based on geographic features (see Materials and Methods). Second, whitefish from VG and LP were morphologically more similar to each other than to fish from SU, indicating that within-lake processes are more important than geographical proximity for explaining the variation in whitefish morphology. Third, an independent origin of profundal specialists recently gained support from a study in a nearby watercourse. Phylogenetic analyses including 17 micro-satellite loci suggested that profundal specialist morphs of whitefish have diverged in sympatry within different lakes (Præbel et al. in review). Taken together, it is most likely that the lakes in this study represent independent replicates of a similar evolutionary process, although confirmation from phylogenetic studies within these lakes is still lacking.

In addition to parallel evolutionary effects, independent populations inhabiting similar environments also frequently show some phenotypic differences, resulting from non-parallel evolution (e.g., Berner et al. 2010; Rosenblum and Harmon 2011; Kaeuffer et al. 2012). We quantified the relative importance of parallel (in response foraging specialization) and non-parallel (lake-specific responses) effects on morphological variation using the framework presented by Langerhans and DeWitt (2004). We found that in terms of magnitude non-parallel evolution between lakes explained a greater proportion (about 45% more) of the overall variation in morphology than the parallel effects of resource specialization. This strongly suggests that either lake-specific selection conditions are shaping the morphological response of the LSR whitefish in different ways in each lake or that whitefish are responding differently and in a non-parallel fashion to the same selection conditions in each lake. However, none of the measured lake characteristics (Table 1) seem to be directly related to these non-parallel differences in fish morphology. Based on physical lake characteristics LP (size) and VG (proportion of available littoral and profundal areas) are the most different lakes, while it was fish from SU that was morphologically most different from the other two lakes (Fig. 4). Despite evidence of a non-parallel divergence effect it is clear that there is also strong parallel morphological divergence. The parallelism in morphology occurs in groups defined by similar ecological traits in different lakes. This strongly supports the hypothesis that divergent natural selection that results from differential specialization on multiple ecological resources is a key process in population divergence and adaptive radiation (Endler 1986; Smith and Skúlason 1996; Schluter 2000).

The parallel morphological effect supports the previously reported ecological divergence of LSR whitefish into distinct littoral and profundal foraging specialists (Siwertsson et al. 2013). Profundal foraging specialists in this study strongly resembled the phenotypes of profundal specialist morphs of whitefish (Kahilainen and Østbye 2006; Harrod et al. 2010) and other fish species (Klemetsen et al. 2002; Zimmerman et al. 2006). This provides strong support for the adaptive significance of the observed morphological characteristics. Whitefish specializing on profundal resources in this study had a more robust body shape, with deep body, a relatively large head compared to body size, large eyes dorsally positioned, and long pectoral fins. This study was based on the expectation that specialization on contrasting resources was the driving factor behind the observed morphological divergence. However, other biotic and abiotic differences between the two habitats may also influence the studied morphological traits. Morphological variation in fish has been described for a wide range of environmental and ecological factors, such as water depth (Zimmerman et al. 2006), water chemistry (Bourgeois et al. 1994; Crispo and Chapman 2011), substrate type (Kristjánsson et al. 2002; Komiya et al. 2011), prey type (Mittelbach et al. 1999), and predation risk (reviewed in Langerhans 2010).

Several of the observed morphological characteristics of the profundal specializing fish have been shown to be of adaptive value for other profundal species. Large eyes are an adaptation to feeding under low light conditions in fish (Pankhurst 1989; Schmitz and Wainwright 2011), and other vertebrates (Hall and Ross 2007; Hall 2008; Schmitz and Motani 2010). Deep body form and ventrally positioned mouth have been suggested as adaptations to feeding near or in the soft sediments in cichlids living in deep water (Genner and Turner 2012). In cichlids, larger head sizes are an indirect effect of increased gill size, correlated to low levels of dissolved oxygen (Langerhans et al. 2007; Crispo and Chapman 2011). It is possible that a similar mechanism may be operating in the reported foraging specialists. The larger caudal region and smaller head and anterior body observed in littoral foraging whitefish specialists are generally thought to be adaptations to predation from piscivorous fish (Langerhans 2010), such as pike (*Esox lucius*) and perch (*Perca fluviatilis*), which is more likely in the littoral compared to the profundal zone. It is likely that the selective forces on body morphology are complex and influenced by multiple biotic and abiotic factors, and that current morphology reflects both contemporary and historical selection pressures (e.g., Langerhans et al. 2007; Spoljaric and Reimchen 2007; Kristjánsson et al. 2011). More comprehensive studies are thus needed to more clearly elaborate which proximate factors actually drive the observed morphological divergence between littoral and profundal specialists. However, there are a number of ultimate effects.

The significant effect of lake on whitefish morphology indicates that genetic differences between whitefish from different lakes may at least partly influence the direction of evolution of morphological traits. However, the observed morphological diversity in whitefish is likely related to both genetic divergence and environmental effects on the developmental processes, i.e., phenotypic plasticity. The relative contribution of these two sources is unknown in the present whitefish populations, but it is likely to vary between different morphological characters and possibly between different populations. In whitefish, gill raker number is a trait considered to have high heritability, with little scope for plastic changes (Svärdson 1952, 1979; Siwertsson et al. 2012). Thus, the documented differences in gill raker number between littoral and profundal specializing whitefish within all three lakes (Table 2; Siwertsson et al. 2013) may be the result of gene frequency change driven by selection. Less is known about the genetic and plastic effects on whitefish body shape and the morphological traits included in this study, but Svärdson (1950) demonstrated an effect of phenotypic plasticity on several measures of head and body proportions related to changes in growth rate. Morphological differences associated to trophic behavior are known to have a genetic basis in several fish species (e.g., McPhail 1984; Skúlason et al. 1989). More specifically, several morphological and behavioral adaptations to profundal resource utilization in Arctic charr have been shown to be genetically determined (Klemetsen et al. 2002, 2006). However, in fish many morphological characters also show strong plastic responses to changes in environment or resource use during the life-time of an individual, and in some cases polymorphism may result primarily from phenotypic plasticity (e.g., Hindar and Jonsson 1993; Mittelbach et al. 1999; Adams and Huntingford 2004). The size and shape of bones and muscles may be greatly modified by the physical process of feeding (Wainwright et al. 1991; Mittelbach et al. 1999), and plasticity may also be particularly pronounced in fish as they show indeterminate growth. In this study, temporal variation in competition and prey availability in profundal and littoral habitats, and the proximity between the two habitats are factors that would favor some levels of phenotypic plasticity. Regardless of the mechanism of divergence, the replicated morphological divergence between littoral and profundal specializing whitefish reflect the importance of divergent natural selection and adaptive (plastic or genetic) differentiation (Winemiller 1991; Johnson and Belk 2001). Probably both genetic divergence and phenotypic plasticity are important in the observed morphological differences between profundal and littoral resource specialists.

The profundal specializing whitefish in this study is as specialized on profundal food and habitat resources as the discrete small body sized profundal whitefish small sparsely rakered (SSR) morph that has been described elsewhere (Harrod et al. 2010; Siwertsson et al. 2010, 2013). The morphological differences between littoral and profundal specializing whitefish reported in this study resemble the morphologies of the clearly separated littoral (LSR) and profundal (SSR) whitefish morphs (Kahilainen and Østbye 2006; Harrod et al. 2010). Possible differences in magnitude of morphological divergence have not been evaluated in this study. However, for gill raker number, the divergence is less pronounced here than between the two discrete whitefish morphs (Kahilainen and Østbye 2006; Harrod et al. 2010; Siwertsson et al. 2010, 2013). Genetic differences between both previously reported littoral and profundal morphs and the specialists included in this study are significant, albeit weak (Siwertsson et al. 2013; Præbel et al. in review). Altogether, our results support the suggestion by Siwertsson et al. (2013) that the large body sized sparsely rakered (LSR) whitefish specialists reported in this study represent an early stage of divergence of two morphs specializing in littoral and profundal foraging resources.

In conclusion, we confirmed the expectations of parallel morphological divergence based on contrasting resource specializations in the studied whitefish populations. However, non-parallel lake-specific morphological characteristics were also evident. The morphology of profundal foraging specialists was similar to profundal specialist morphs of whitefish and other fish species, which suggest an adaptive significance of the recorded morphological traits. Furthermore, this study, showing similar phenotypic adaptations to similar environments, supports the suggestion from Siwertsson et al. (2013) of ecological and morphological divergence within the LSR whitefish morph, possibly leading to the formation of two discrete benthic specialist whitefish morphs.

## Appendix

**Table A1:** Effects of lake, foraging specialization (Spec.), and their interaction on the first three PC-axes from PCAs of body shape and linear trait measurements, estimated using two-way ANOVA

			Spec.		Lake		Spec. × Lake	
					
		Variance Explained (%)	*F*	*P*-value	*F*	*P*-value	*F*	*P*-value
Body shape	PC 1	28.5	21.9	<0.001	5.8	<0.01	0.3	0.72
	PC 2	17.0	17.4	<0.001	91.9	<0.001	0.3	0.76
	PC 3	14.7	24.7	<0.001	1.6	0.20	2.1	0.12
Linear traits	PC 1	59.2	11.6	<0.001	77.1	<0.001	0.1	0.87
	PC 2	14.7	75.6	<0.001	14.2	<0.001	0.6	0.52
	PC 3	8.2	16.4	<0.001	50.1	<0.001	9.7	<0.001

**Table A2:** Loadings (importance) of different linear traits on the first three PC-axes. The three most important morphological traits are in boldface

Morphological trait	PC1	PC2	PC3
Eye diameter	**−0.31**	0.21	**0.50**
Snout length	**−0.77**	**−0.34**	0.01
Maxilla length	**−0.43**	0.14	**−0.63**
Head length	**−**0.30	0.11	0.09
Head depth	**−**0.12	0.20	0.01
Body depth anterior	0.06	0.29	**−**0.21
Body depth posterior	0.05	0.18	**−**0.25
Caudal peduncle depth	**−**0.01	0.18	**−**0.24
Dorsal fin length	**−**0.02	**0.48**	**−**0.23
Pectoral fin length	**−**0.15	**0.63**	**0.37**

## References

[b1] Adams CE, Huntingford FA (2004). Incipient speciation driven by phenotypic plasticity? Evidence from sympatric populations of Arctic charr. Biol. J. Linn. Soc.

[b2] Adams DC, Rohlf FJ, Slice DE (2004). Geometric morphometrics: ten years of progress following the ‘revolution’. It. J. Zool.

[b3] Amundsen P-A (1988). Habitat and food segregation of two sympatric populations of whitefish (*Coregonus lavaretus* L. *s.l*.) in Stuorajavri, northern Norway. Nord. J. Freshw. Res.

[b4] Amundsen P-A, Bøhn T, Våga GH (2004a). Gill raker morphology and feeding ecology of two sympatric morphs of European whitefish (*Coregonus lavaretus*. Ann. Zool. Fenn.

[b5] Amundsen P-A, Knudsen R, Klemetsen A, Kristoffersen R (2004b). Resource competition and interactive segregation between sympatric whitefish morphs. Ann. Zool. Fenn.

[b6] Arnold SJ (1983). Morphology, performance and fitness. Am. Zool.

[b7] Bergstrand E (1982). The diet of four sympatric whitefish species in Lake Parkijaure. Rep. Inst. Freshw. Res. Drottningholm.

[b8] Bernatchez L, Hendry AP, Stearns SC (2004). Ecological theory of adaptive radiation. An empirical assessment from coregonine fishes (Salmoniformes). Evolution illuminated, salmon and their relatives.

[b9] Berner D, Roesti M, Hendry AP, Salzburger W (2010). Constraints on speciation suggested by comparing lake-stream stickleback divergence across two continents. Mol. Ecol.

[b10] Bourgeois JF, Blouw DM, Koenings JP, Bell MA (1994). Multivariate-analysis of geographic covariance between phenotypes and environments in the threespine stickleback, *Gasterosteus aculeatus*, from the Cook Inlet Area, Alaska. Can. J. Zool.

[b11] Crispo E, Chapman LJ (2011). Hypoxia drives plastic divergence in cichlid body shape. Evol. Ecol.

[b12] Endler JA (1986). Natural selection in the wild.

[b13] Eroukhmanoff F, Hargeby A, Arnberg NN, Hellgren O, Bensch S, Svensson EI (2009). Parallelism and historical contingency during rapid ecotype divergence in an isopod. J. Evol. Biol.

[b14] Genner MJ, Turner GF (2012). Ancient hybridization and phenotypic novelty within Lake Malawi's cichlid fish radiation. Mol. Biol. Evol.

[b15] Gowell C, Quinn T, Taylor EB (2012). Coexistence and origin of trophic ecotypes of pygmy whitefish, *Prosopium coulterii*, in southwestern Alaskan lake. J. Evol. Biol.

[b16] Hall MI (2008). Comparative analysis of the size and shape of the lizard eye. Zoology.

[b17] Hall MI, Ross CF (2007). Eye shape and activity pattern in birds. J. Zool.

[b18] Hammer Ø, Harper DAT, Ryan PD (2001). PAST: paleontological statistics software package for education and data analysis. Paleontologia Electronica.

[b19] Harrod C, Mallela J, Kahilainen KK (2010). Phenotype-environment correlations in a putative whitefish adaptive radiation. J. Anim. Ecol.

[b20] Hindar K, Jonsson B (1993). Ecological polymorphism in Arctic charr. Biol. J. Linn. Soc.

[b21] Johnson JB, Belk MC (2001). Predation environment predicts divergent life-history phenotypes among populations of the livebearing fish *Brachyrhaphis rhabdophora*. Oecologia.

[b22] Jones R, Culver DC, Kane TC (1992). Are parallel morphologies of cave organisms the result of similar selection pressures. Evolution.

[b23] Kaeuffer R, Peichel CL, Bolnick DI, Hendry AP (2012). Parallel and nonparallel aspects of ecological, phenotypic, and genetic divergence across replicate population pairs of lake and stream stickleback. Evolution.

[b24] Kahilainen K, Østbye K (2006). Morphological differentiation and resource polymorphism in three sympatric whitefish *Coregonus lavaretus* (L.) forms in a subarctic lake. J. Fish Biol.

[b25] Kahilainen K, Malinen T, Tuomaala A, Lehtonen H (2004). Diel and seasonal habitat and food segregation of three sympatric *Coregonus lavaretus* forms in a subarctic lake. J. Fish Biol.

[b26] Kahilainen KK, Siwertsson A, Gjelland KØ, Knudsen R, Bøhn T, Amundsen P-A (2011). The role of gill raker number variability in adaptive radiation of coregonid fish. Evol. Ecol.

[b27] Klemetsen A, Elliott JM, Knudsen R, Sørensen P (2002). Evidence for genetic differences in the offspring of two sympatric morphs of Arctic charr. J. Fish Biol.

[b28] Klemetsen A, Knudsen R, Primicerio R, Amundsen P-A (2006). Divergent, genetically based feeding behaviour of two sympatric Arctic charr, *Salvelinus alpinus* (L.), morphs. Ecol. Freshw. Fish.

[b29] Klingenberg CP, Marcus LF, Corti M, Loy A, Naylor G, Slice DE (1996). Multivariate allometry. Advances in morphometrics.

[b30] Klingenberg CP (2011). MorphoJ: an integrated software package for geometric morphometrics. Mol. Ecol. Resour.

[b31] Komiya T, Fujita S, Watanabe K (2011). A novel resource polymorphism in fish, driven by differential bottom environments: an example from an ancient lake in Japan. PLoS ONE.

[b32] Kristjánsson BK, Skúlason S, Noakes DLG (2002). Morphological segregation of Icelandic threespine stickleback (*Gasterosteus aculeatus* L). Biol. J. Linn. Soc.

[b33] Kristjánsson BK, Malmquist HJ, Ingimarsson F, Antonsson T, Snorrason SS, Skúlason S (2011). Relationships between lake ecology and morphological characters in Icelandic Arctic charr, *Salvelinus alpinus*. Biol. J. Linn. Soc.

[b34] Langerhans RB (2010). Predicting evolution with generalized models of divergent selection: a case study with Poeciliid fish. Integr. Comp. Biol.

[b35] Langerhans RB, DeWitt TJ (2004). Shared and unique features of evolutionary diversification. Am. Nat.

[b36] Langerhans RB, Chapman LJ, DeWitt TJ (2007). Complex phenotype-environment associations revealed in an East African cyprinid. J. Evol. Biol.

[b37] Losos JB (2009). Lizards in an evolutionary tree: ecology and adaptive radiation of Anoles.

[b38] McPhail JD (1984). Ecology and evolution of sympatric sticklebacks (*Gasterosteus*) - morphological and genetic evidence for a species pair in Enos Lake, British Columbia. Can. J. Zool.

[b39] Mittelbach GC, Osenberg CW, Wainwright PC (1999). Variation in feeding morphology between pumpkinseed populations: phenotypic plasticity or evolution?. Evol. Ecol. Res.

[b40] Østbye K, Bernatchez L, Næsje TF, Himberg KJM, Hindar K (2005a). Evolutionary history of the European whitefish *Coregonus lavaretus* (L.) species complex as inferred from mtDNA phylogeography and gill-raker numbers. Mol. Ecol.

[b41] Østbye K, Næsje TF, Bernatchez L, Sandlund OT, Hindar K (2005b). Morphological divergence and origin of sympatric populations of European whitefish (*Coregonus lavaretus* L.) in Lake Femund, Norway. J. Evol. Biol.

[b42] Pankhurst NW (1989). The relationship of ocular morphology to feeding modes and activity periods in shallow marine teleosts from New Zealand. Environ. Biol. Fishes.

[b43] Præbel K, Knudsen R, Siwertsson A, Kahilainen KK, Østbye K, Peruzzi S Ecological speciation in postglacial European whitefish: rapid adaptive radiations into the littoral, pelagic and profundal lake habitats. Mol. Ecol.

[b44] R Development Core Team (2011). R: a language and environment for statistical computing.

[b45] Rohlf FJ (2010). TpsDig v2.16.

[b46] Rosenblum EB, Harmon LJ (2011). “Same same but different”: replicated ecological speciation at White Sands. Evolution.

[b47] Sanderson SL, Cheer AY, Goodrich JS, Graziano JD, Callan WT (2001). Crossflow filtration in suspension-feeding fishes. Nature.

[b48] Schluter D (2000). The ecology of adaptive radiation.

[b49] Schmitz L, Motani R (2010). Morphological differences between the eyeballs of nocturnal and diurnal amniotes revisited from optical perspectives of visual environments. Vision. Res.

[b50] Schmitz L, Wainwright PC (2011). Nocturnality constrains morphological and functional diversity in the eyes of reef fishes. BMC Evol. Biol.

[b51] Senar JC, Lleonart J, Metcalfe NB (1994). Wing-shape variation between resident and transient wintering Siskins *Carduelis spinus*. J. Avian Biol.

[b52] Siwertsson A, Knudsen R, Kahilainen KK, Præbel K, Primicerio R, Amundsen P-A (2010). Sympatric diversification as influenced by ecological opportunity and historical contingency in a young species lineage of whitefish. Evol. Ecol. Res.

[b53] Siwertsson A, Knudsen R, Amundsen P-A (2012). Temporal stability in gill raker numbers of subarctic European whitefish populations. Adv. Limnol.

[b54] Siwertsson A, Knudsen R, Præbel K, Adams CE, Newton J, Amundsen P-A (2013). Discrete foraging niches promote ecological, phenotypic and genetic divergence in sympatric whitefish (*Coregonus lavaretus*. Evol. Ecol.

[b55] Skúlason S, Smith TB (1995). Resource polymorphisms in vertebrates. Trends Ecol. Evol.

[b56] Skúlason S, Noakes DLG, Snorrason SS (1989). Ontogeny of trophic morphology in four sympatric morphs of arctic charr *Salvelinus alpinus* in Thingvallavatn, Iceland. Biol. J. Linn. Soc.

[b57] Smith TB, Skúlason S (1996). Evolutionary significance of resource polymorphisms in fishes, amphibians, and birds. Annu. Rev. Ecol. Syst.

[b58] Spoljaric MA, Reimchen TE (2007). 10 000 years later: evolution of body shape in Haida Gwaii three-spined stickleback. J. Fish Biol.

[b59] Svärdson G (1950). The coregonid problem. II. Morphology of two coregonid species in different environments. Rep. Inst. Freshw. Res. Drottningholm.

[b60] Svärdson G (1952). The coregonid problem. IV. The significance of scales and gillrakers. Rep. Inst. Freshw. Res. Drottningholm.

[b61] Svärdson G (1979). Speciation of Scandinavian *Coregonus*. Rep. Inst. Freshw. Res. Drottningholm.

[b62] Turgeon J, Estoup A, Bernatchez L (1999). Species flock in the North American Great Lakes: molecular ecology of Lake Nipigon ciscoes (Teleostei: Coregonidae: *Coregonus*. Evolution.

[b63] Wainwright PC, Wainwright PC, Reilly SM (1994). Functional morphology as a tool in ecological research. Ecological morphology: integrative organismal biology.

[b64] Wainwright PC (1996). Ecological explanation through functional morphology: the feeding biology of sunfishes. Ecology.

[b65] Wainwright PC, Osenberg CW, Mittelbach GG (1991). Trophic polymorphism in the pumpkinseed sunfish (*Lepomis gibbosus* Linnaeus): effects of environment on ontogeny. Funct. Ecol.

[b66] Wimberger PH, Stouder DJ, Fresh K, Feller RJ (1994). Trophic polymorphisms, plasticity, and speciation in vertebrates. Theory and application in fish feeding ecology.

[b67] Winemiller KO (1991). Ecomorphological diversification in lowland fresh-water fish assemblages from five biotic regions. Ecol. Monogr.

[b68] Zelditch ML, Swiderski DL, Sheets HD, Fink WL (2004). Geometric morphometrics for biologists: a primer.

[b69] Zimmerman MS, Krueger CC, Eshenroder RL (2006). Phenotypic diversity of lake trout in Great Slave Lake: differences in morphology, buoyancy, and habitat depth. Trans. Am. Fish. Soc.

